# Parapharyngeal Abscesses as a Posttonsillectomy Complication

**DOI:** 10.7759/cureus.64826

**Published:** 2024-07-18

**Authors:** Mohamed Elmarghani

**Affiliations:** 1 Ear, Nose, and Throat, St John's Hospital, National Health Service (NHS) Scotland, Edinburgh, GBR

**Keywords:** posttonsillectomy complication, early postoperative complication, peritonsillar, deep neck space infection, parapharyngeal abscess

## Abstract

We present a rare case involving a patient in his 50s who developed a parapharyngeal abscess two days following an elective tonsillectomy. Despite being rare, deep neck space infections have been increasingly reported in patients following tonsillectomies. This report explores their prevalence, risk factors, and potential complications and outlines appropriate management strategies.

## Introduction

Tonsillectomies in the United Kingdom are often elective routine procedures indicated for various conditions, such as recurrent tonsillitis and obstructive sleep apnoea. They offer significant symptomatic relief and are generally associated with positive outcomes. However, like any surgical intervention, it carries potential risks and complications that can affect recovery. These range from common issues such as pain and bleeding to more severe problems like deep neck space infections.

A large retrospective cohort study in Taiwan, which included all patients who underwent tonsillectomy between 2001 and 2009, found that tonsillectomised patients had a 1.71-fold greater risk of developing deep neck infection (DNI). The study analysed 9,915 patients with tonsillectomies and 99,150 in a comparison cohort. After matching for sex and age, it was found that 34 out of 9,915 tonsillectomised patients developed DNI (71.6 per 100,000 person-years), compared to 174 individuals in the control cohort (36.6 per 100,000 person-years). The overall relative risk of DNI in the tonsillectomy cohort was 2.0 (95% CI, 1.4-2.8). The increased risk of developing DNI was significant in both sexes and in patients younger than 40 years old [1].

These infections are particularly dangerous due to their potential for rapid progression and impact on critical neck structures, such as the airway, major blood vessels, and nerves. Early detection and treatment are vital to avert severe complications like airway obstruction, sepsis, or mediastinitis. For healthcare providers, understanding the risk factors, symptoms, and approach strategies for deep neck space infections following a tonsillectomy is crucial to delivering prompt and effective patient care [2].

## Case presentation

A 56-year-old male came to the hospital for an elective tonsillectomy due to repeated tonsillitis episodes. He was previously fit and well, with the last tonsillitis episode occurring six months ago. He has no background medical conditions (including diabetes mellitus [DM]) or known allergies. He never smokes and only drinks around two units of alcohol a week. He works in a supermarket as a cashier and resides with his wife and children. He has no history of dental infections.

The elective tonsillectomy was uneventful, with no intraoperative complications. He was well postoperatively and was discharged later in the day with regular analgesia and general advice. He was not sent home with antibiotics.

His first day postoperatively was uneventful, and the patient felt that the pain was manageable with regular analgesia. Overnight, however, he developed fevers, regular coughing, and an increase in his pain. The next morning, he noticed some difficulty in swallowing. Later in the afternoon, he reported further episodes of fever, coughed up white metallic-tasting sputum, had a complete inability to swallow, and had worsening pain. He presented to the accident and emergency department later that evening.

On examination, the patient appeared clinically dehydrated and pyrexic. He had an evident left-sided neck swelling with a marked reduction in neck movement. Looking at the oral cavity, a white slough consistent with a normal posttonsillectomy appearance was noted, and no obvious abnormalities were detected. There was also no evidence of any dental infection.

His blood laboratory results demonstrated markedly raised inflammatory markers with a neutrophil-dominant white blood cell count of 22 × 10^9^/L and a C-reactive protein level of 232. His blood glucose was 5.0 mmol/L, which is well within the normal limits. A flexible nasoendoscopy showed significant parapharyngeal swelling with pooling of pus above the glottis and poorly visible vocal cords. There was no evidence of sinusitis. Unfortunately, the images were lost, and we could not attach them to this report.

He received stat IV doses of ceftriaxone 2 g, metronidazole 500 mg, dexamethasone 6.6 mg, and adrenaline nebulisers. The anaesthesiology team was requested to evaluate the patient, and a decision was made to proceed with awake fiber-optic intubation. Swabs were planned to be taken intraoperatively.

**Figure 1:**
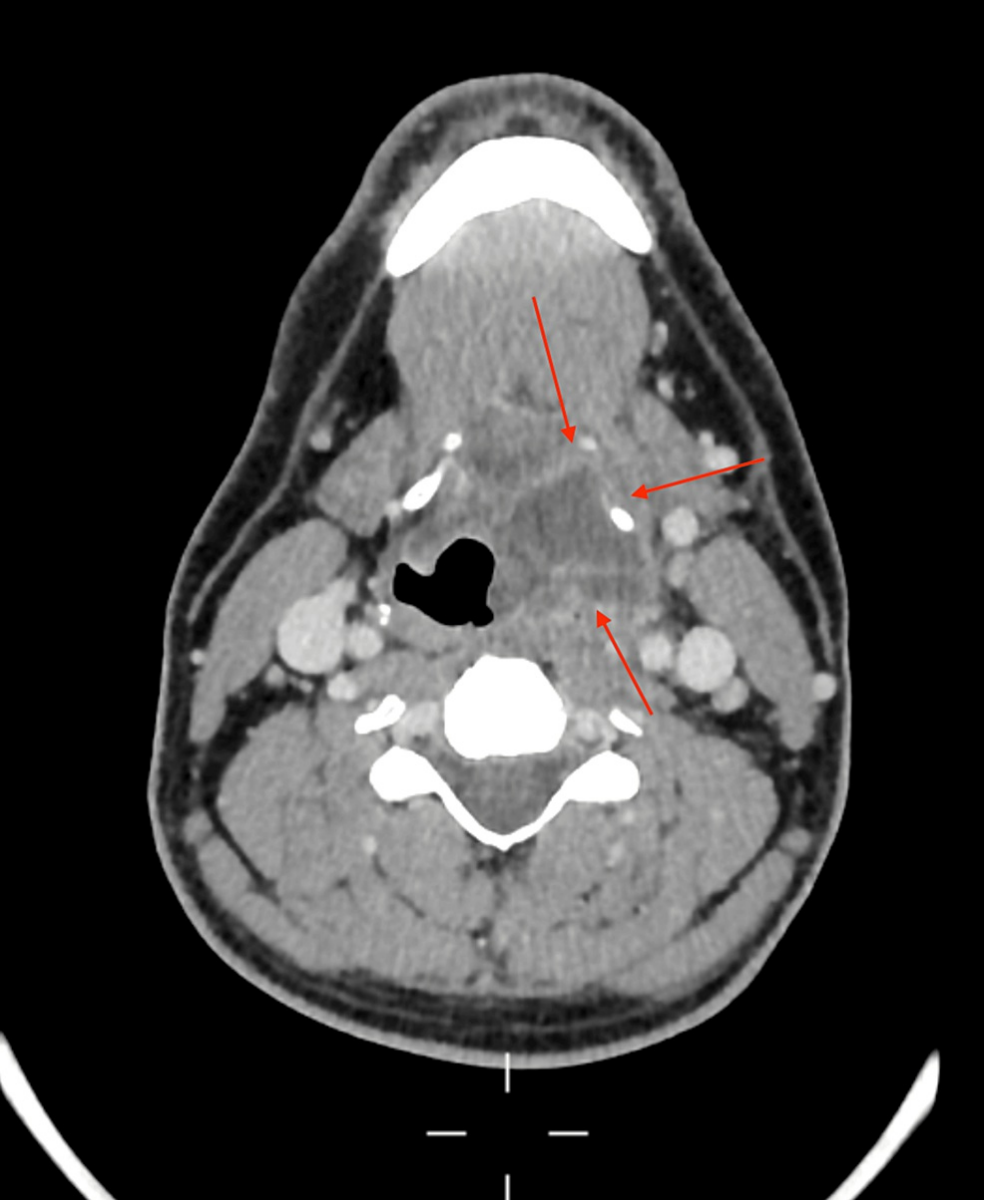
Axial view of a CT scan of the neck at the level of C1/C2 demonstrating a collection behind the left tonsillar fossa (red arrows)

**Figure 2:**
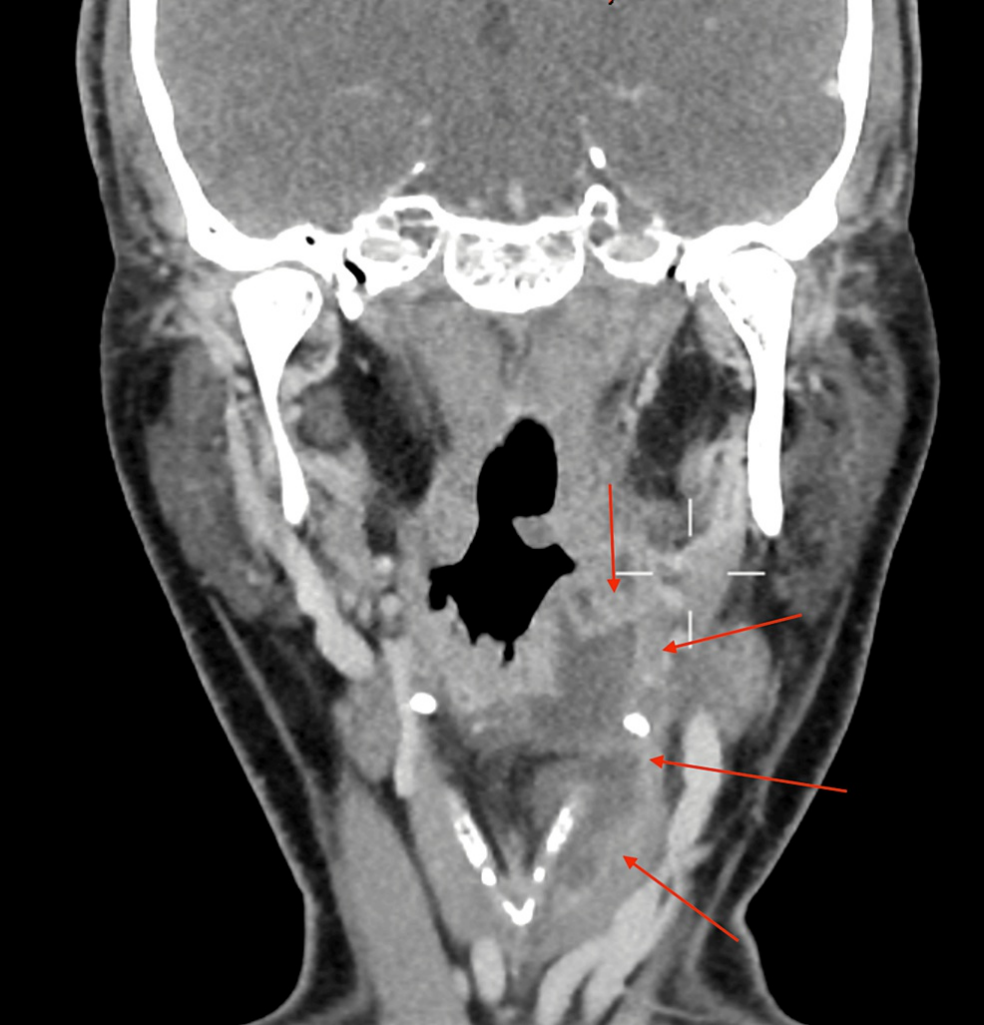
Coronal view of a CT scan of the neck demonstrating a left-sided collection extending from the glottis to the thyroid cartilage and between the strap muscles (red arrows)

**Figure 3:**
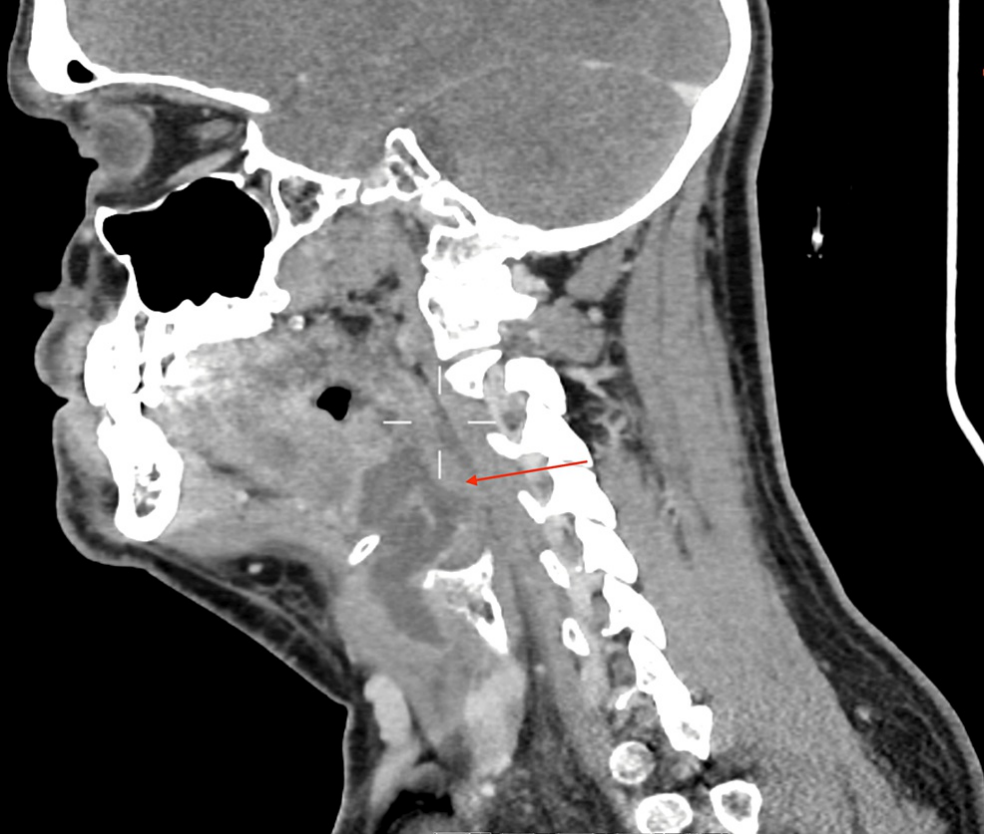
Sagittal view of a CT neck demonstrating a left-sided parapharyngeal collection (red arrow)

He then had a CT scan that demonstrated a large collection from the left faucial tonsil bed into the preepiglottic fat and superficially between the thyroid cartilage and strap muscles down to the glottis level, as shown in Figures 1-3. There was no evidence of a collection in the chest.

The patient was subsequently taken to the theater approximately one hour after presentation to the accident and emergency department for a direct laryngo-pharyngoscopy, intraoral drainage, and external exploration and drainage of the left parapharyngeal abscess with nasogastric (NG) tube insertion. Pus was collected and sent for cultures. The results indicated the presence of group A Streptococcus.

The antibiotics of choice, under microbiology guidance, involved ceftriaxone 2 g once daily and metronidazole 500 mg three times daily for beta-lactamase and anaerobic organism coverage, which are often the culprit organisms. He received a stat dose before the surgery and continued taking them postoperatively as the swabs showed sensitivity.

He was transferred to the intensive care unit postoperatively and continued on broad-spectrum intravenous antibiotics. He recovered well with no other complications and was extubated 24 hours later. He was transferred to the ward and had his NG tube eventually removed once he was able to eat and drink. He was discharged two days later with a follow-up nasal endoscopy planned in a week's time.

## Discussion

Posttonsillectomy abscesses, although rare, represent significant complications necessitating prompt recognition and intervention due to their potential for rapid progression and severe consequences. Most studies and clinical observations indicate that the highest risk period for infections is between days 5 and 10 postoperation, which makes our case fairly unusual [2].

Several factors likely contribute to the development of these abscesses, which can be categorised into patient-related and non-patient-related factors. Patient-related factors include poor oral hygiene, trauma, and immunocompromised status, such as intravenous drug use, DM, and chronic steroid use. In pediatric cases, additional causes include infection of a second branchial cleft fistula and Weber's glands, and dental disease [3].

Non-patient-related factors encompass operative issues, such as incomplete removal of tonsillar tissue and inadequate sterilisation of surgical instruments. It has also been suggested that tonsillectomy induces local immunological changes predisposing patients to localised infections [3]. Furthermore, according to a comparative study, hot tonsillectomies have been shown to pose a higher risk for postoperative infections, including deep neck space infections, due to thermal injury to surrounding tissues. The use of heat can lead to more extensive tissue damage and a greater potential for infection [4]. In our case, this is the only risk factor the patient had.

Diagnosis is predominantly clinical, aided by flexible nasoendoscopy to visualise parapharyngeal swelling. Imaging studies, such as contrast-enhanced CT scans of the neck and chest, are essential to exclude other complications and guide surgical management. Potential complications include airway obstruction necessitating early anaesthetic assessment, internal jugular vein thrombosis, necrotising fasciitis of the neck, mediastinitis, septic shock, and death [5].

Management strategies vary based on the patient's clinical status and the extent of the abscess. In cases where the patient's condition is stable, the abscess is relatively small, and major complications are ruled out, a conservative approach involving intravenous antibiotics and close monitoring may be sufficient. However, in more severe cases, surgical intervention with incision, drainage, and washout, combined with broad-spectrum intravenous antibiotics, is the preferred approach.

The external approach typically uses either a modified apron incision or a hockey-stick incision. To access the parapharyngeal space, the sternocleidomastoid muscle is retracted posteriorly using blunt dissection, which protects the internal jugular vein and carotid arteries. The abscess cavity is then entered and drained.

The internal, or intraoral, method involves making a longitudinal incision across the pharyngeal wall to drain the abscess. When the patient is under general anaesthesia, the Trendelenburg position is used to prevent aspiration. This procedure can also be performed under local anaesthesia if the abscess is easily accessible, though it requires significant patient cooperation. Intraoral drainage offers advantages such as less morbidity, shorter hospital stays, and lower costs. However, it is considered less aggressive compared to external drainage and is not suitable for abscesses located lateral to large vessels or those involving multiple compartments [6].

Radiologically guided drainage has also been documented as an effective alternative for managing these abscesses. A randomised controlled trial in 2013 demonstrated that ultrasound-guided drainage of deep neck space abscesses in certain patients is effective and safe, and results in significant healthcare cost savings [7]. Postoperatively, patients should be initially monitored in a high-dependency unit before being transferred to a general ward.

## Conclusions

Posttonsillectomy abscess collections represent a serious complication with the potential to be life-threatening. Clinicians must maintain a high index of suspicion when evaluating patients presenting with postoperative symptoms such as pain and fever, avoiding the premature dismissal of these signs. Despite their rarity, the morbidity and mortality associated with undiagnosed deep neck space infections are significant.

To minimise complications, a combination of thorough clinical examination and investigations relating to risk factors, early involvement of an anaesthetic team, and meticulous surgical planning with a well-defined postoperative management plan is essential.

.
